# Editorial: Personalized Precision Medicine in Autism Spectrum-Related Disorders

**DOI:** 10.3389/fneur.2021.730852

**Published:** 2021-09-28

**Authors:** Lidia V. Gabis, Raz Gross, Josephine Barbaro

**Affiliations:** ^1^Department of Pediatrics, Sackler School of Medicine, Tel Aviv University, Tel Aviv, Israel; ^2^Maccabi Health Care Services, Tel Aviv, Israel; ^3^Sheba Medical Center, Ramat Gan, Israel; ^4^School of Psychology and Public Health, La Trobe University, Melbourne, VIC, Australia

**Keywords:** autism spectrum disorder, genetics, child, infant, medical, personalized precision medicine

According to the National institute of Health (NIH), the definition of Personalized Precision Medicine is to enable risk assessment, diagnosis, prevention, and therapy specifically tailored to the unique characteristics of the individual, thus enhancing quality of life and public health. This concept has been adopted in all areas of medicine, acknowledging individual differences. Despite the shared features defining the autism spectrum disorder (ASD) phenotype, as listed in Diagnostic and Statistical Manual of Mental Disorder-5 (DSM-5) (1), a more personalized and precise approach is currently recommended for individuals with ASD (2, 3).

Individual differences span from etiology and early life differences to specific developmental trajectories according to gender, age of diagnosis, and severity, and up to response to treatment (4, 5). The clinical heterogeneity of ASD in terms of etiology, symptoms, severity, and response to treatment is demonstrated in the current issue.

In terms of developmental history and trajectory, individuals with ASD may have been diagnosed in childhood, adolescence, or as adults, and clearly the clinical presentation and severity may differ according to age of diagnosis. For children experiencing developmental differences early in life, the main aim is to recognize early signs and symptoms to be able to provide early intervention, enhancing and promoting positive lifelong outcome. In the important overview of “Autism Screening in Early Childhood,” Brewer et al. provide practical tools for evaluation and implementation of ASD screeners and provide insights for developing reliable screening instruments. One of the screeners covered in this special issue is seen in Barbaro et al.'s study [A Pilot Investigation of the Social Attention and Communication Surveillance (SACS) Tool for the Early Identification of Autism in Tianjin, China]. They implemented the SACS-C screening tool for a large population of children in Tianjin, which was shown to be more sensitive and reliable than the Checklist for Autism in Toddlers-23 (CHAT-23) for identification of ASD in 1–2-year-olds, after training for both instruments was implemented, and children followed after age 3. Training on SACS-C was recommended for screening large populations of young children in China.

As symptoms of autism often appear during the 1st year of life, Gabis et al. revealed an early sign that may be easily recognized, measured, and serve as a “red flag” to prompt a structured diagnostic evaluation and diagnosis of autism. This study focuses on early motor differences and points to hypotonia (low muscle tone) as a sign to prompt ASD diagnosis and enable earlier recognition in infants at risk. Gender differences were demonstrated in initial presentation, emphasizing the need for increased awareness of ASD in girls.

Early differences in language development was addressed by Oren et al. Acquisition of language in toddlers with ASD was considerably later as compared to typically developing peers. However, the encouraging finding was that while toddlers with ASD were late in producing first words, the accumulation pace of new words became similar.

The formal diagnosis of ASD requires clinical expertise and performance of lengthy “gold standard” tools, which can lead to a suboptimal rate of diagnosis. The significant delays in age of diagnosis and discrepancies in availability of diagnostic tools, intervention services, and in the level of professional expertise are a significant challenge in Brazil, as described by Sukiennik et al. The identification of ASD is prerequired for provision of appropriate intervention and treatment. In addition, the flourishing of clinical studies examining specific treatments and outcomes posed a new challenge of applying an objective, reliable, and reproducible measure of assessment and measure of improvement, since the current methods rely mainly on behavioral measures that are subjective, less reliable, and influenced by the “placebo effect.” We hypothesize that the failure of most ASD behavioral and pharmacological therapy trials is mainly due to mixing biologically different ASD populations, as well as focusing on subjective measures of behavioral changes as endpoints for the studies, measures that are strongly influenced by placebo response. It is possible that some of the drugs or behavioral interventions could have had significant benefits in a more biologically homogenous sub-population of ASD and by using a more reliable and objective biomarker for measuring change.

Tsuchiya et al., in their paper from Japan: “Diagnosing Autism Spectrum Disorder Without Expertise: A Pilot Study of 5- to 17-Year-Old Individuals Using Gazefinder” suggest an easy to use and reliable tool that can be used as a screener for identification of ASD risk, before the recommendation of full expert assessment. This eye tracking tool is easy to use on a standard computer and monitor, the examination takes <2 min, the algorithm was accurate in identifying ASD vs. non-ASD with 78% accuracy on the 98% of participants who succeeded in completing the task. As such, this tool may serve as a screener as well as a marker to monitor improvement in ASD symptomatology (eye gaze).

ASDs are extremely variable in developmental trajectory, severity, and co-occurring conditions (across the lifespan), such as sleep difficulties in infancy, Attention Deficit Hyperactivity Disorder (ADHD) in childhood, and mood disorders in adolescence, as well as other co-occurring disorders such as dysmorphism, epilepsy, or intellectual disability. There is often also variation in how different patients are treated, as some may be treated for their core symptoms of communication difficulties and atypical behavior, and others for their comorbidities. Neurological comorbidities such as epilepsy, motor impairments, and abnormal brain growth or malformations (including macrocephaly, absence of the corpus callosum, or migration defects) may become a hint for specific genetic etiology such as PTEN (macrocephaly), SCN1A (Dravet epileptic syndrome), Tuberous Sclerosis (tumors), and Angelman's syndrome (sleep disturbance, microcephaly, and motor impairment). The extensive research into the most common inherited disorder causing ASD and Intellectual disability, Fragile X Syndrome, led to multiple research venues of treatments. One of the paths is reviewed by Rajaratnam et al. who assessed the response to Sertraline (Selective serotonin reuptake inhibitor) in Fragile X patients as compared to non-syndromic ASD, and found a specific and differential response to this treatment, emphasizing that despite of the shared ASD phenotype, only the Fragile X group with the comorbidity of anxiety responded to this treatment.

Gozes' review, “The ADNP Syndrome and CP201 (NAP) Potential and Hope,” demonstrates the path leading from identification of specific clinical features including dysmorphism, motor delay, and cognitive impairments in children with ASD, and performed genetic testing to search for a specific etiology of ASD. In this case, the very rare ADNP mutation was found by exome sequencing, and research into the mechanism of action may bring hope for specific treatment and for a potential cure in ADNP syndrome patients, and possibly in additional related causes of autism, since ADNP was found to be a regulatory gene of more than 400 genes critical for brain development. A drug candidate, CP201 (NAP), for intranasal administration was developed and examined in preclinical studies for amelioration of symptoms resembling ASD in a mouse model of ADNP deficiency.

Hu and Bi approached the genetic differences in ASD from a different perspective, by analyzing phenotypic differences in view of transcriptomic data as related to specific ASD-associated genes, using Weighted Gene Correlation Network Analyses (WGCNA). Children from simplex and multiplex families were divided into phenotypic categories and differential gene expression analysis was performed, showing that phenotypic subtyping improved the ability to discern between probands and typically developing siblings in simplex families.

Susceptibility to ASD is now understood to be partially due to rare genetic variants, and partially due to environmental factors including prenatal viruses, perinatal brain insult, and premature birth. Prematurity is associated with ASD symptomatology in more than 7% of children born premature, and the risk increases with each additional week of prematurity (6). The study “A Comparison of Children Born Preterm and Full-Term on the Autism Spectrum in a Prospective Community Sample” by Luu et al., comparing children with ASD born before term to children with ASD born at term, did not reveal significant differences in visual reception, fine motor, receptive and expressive language, and autism behaviors, demonstrating that ASD impact is similar regardless of prematurity risk factors, which differed to that of most previous research; however, a large proportion of the sample was in the moderate to late preterm group, which is less impacted by disability than extreme prematurity.

Language, communication, and behavioral brain pathways are complex and vulnerable. There are many factors that cause similar features, signs, and symptoms presenting as an ASD phenotype. Some pathways may have a common response to treatment and intervention, especially during early development where there is increased brain plasticity, driving the research toward earlier identification of atypical trajectories. Other treatments in clinical research focus on individual differences and attempt to tackle specific autism-related pathways to circumvent the shared features and address the specific underlying disorder. Challenges and innovative individualized approaches to autism are presented in the following 14 papers, which encompass studies from diverse research groups all around the world.

## Author Contributions

All authors listed have made a substantial, direct and intellectual contribution to the work, and approved it for publication.

## Conflict of Interest

The authors declare that the research was conducted in the absence of any commercial or financial relationships that could be construed as a potential conflict of interest.

## Publisher's Note

All claims expressed in this article are solely those of the authors and do not necessarily represent those of their affiliated organizations, or those of the publisher, the editors and the reviewers. Any product that may be evaluated in this article, or claim that may be made by its manufacturer, is not guaranteed or endorsed by the publisher.
